# Spin Circuit Model for 2D Channels with Spin-Orbit Coupling

**DOI:** 10.1038/srep20325

**Published:** 2016-03-02

**Authors:** Seokmin Hong, Shehrin Sayed, Supriyo Datta

**Affiliations:** 1 School of Electrical and Computer Engineering, Purdue University, IN, 47907, USA

## Abstract

In this paper we present a general theory for an arbitrary 2D channel with “spin momentum locking” due to spin-orbit coupling. It is based on a semiclassical model that classifies all the channel electronic states into four groups based on the sign of the *z*-component of the spin (up (*U*), down (*D*)) and the sign of the *x*-component of the velocity (+, −). This could be viewed as an extension of the standard spin diffusion model which uses two separate electrochemical potentials for *U* and *D* states. Our model uses four: *U*+, *D*+, *U*−, and *D*−. We use this formulation to develop an equivalent spin circuit that is also benchmarked against a full non-equilibrium Green’s function (NEGF) model. The circuit representation can be used to interpret experiments and estimate important quantities of interest like the charge to spin conversion ratio or the maximum spin current that can be extracted. The model should be applicable to topological insulator surface states with parallel channels as well as to other layered structures with interfacial spin-orbit coupling.

Recently there have been a number of electrical measurements showing the unique coupling between charge and spin in the surface states of a new class of materials called topological insulators (TI)^1^,^2^,^3^,^4^,^5^,^6^,^7^,^8^,^9^,^10^,^11^,^12^. These include charge current induced spin voltage as well as spin current induced charge voltage analogous to the spin Hall effect (SHE) and the inverse spin Hall effect (ISHE) respectively that are observed in a different class of materials with strong spin-orbit coupling (see, for example, references in^13^,^14^). Although there might be some fundamental differences in their physical origin there are irrefutable similarities regarding terminal characteristics of charge and spin in these two classes of materials. The latter phenomena are usually interpreted in terms of a bulk spin diffusion equation modified to include the spin Hall angle^15^,^16^ which often coexist with other spin-orbit torques like interfacial Rashba-style spin-orbit coupling (SOC)^17^ similar to those observed earlier in semiconductors (see ref. 18 and references therein). With successful demonstrations of writing information into a conventional metallic magnet at room temperature using materials with SHE there is also intense theoretical interest and discussion^19^,^20^,^21^,^22^,^23^,^24^,^25^ in understanding the physics and implications of this type of effect in TI with particular interest in their capability of spin current generation.

In this paper we present a general theory for an arbitrary 2D channel in the *z*-*x* plane with SOC (Fig. 1(a)) of the form 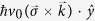 that gives rise to electronic states whose *x*-directed momentum and *z*-directed spins are correlated. We present a semiclassical model based on which we develop an equivalent spin circuit that can be used to interpret experiments and estimate important parameters of great interest like the charge to spin conversion ratio or the maximum spin current density that can be extracted. Note that a primary value of the modular circuit approach is that it allows us to characterize the TI conductor based on its intrinsic properties irrespective of what the terminals are connected to. Our formulation includes many spin transport related effects through the use of 4-component (one for charge, three for spin) voltages and currents. This “spin circuit” approach has been described in several earlier publications and benchmarked against experiment as well as against diffusion theory and quantum transport models (see, for example, ref. 26 and references therein).

Our model is based on a classification of all electronic states in the channel into four groups based on the sign of the *z*-component of the spin (up (*U*), down (*D*)) and the sign of *x*-component of the velocity (+, −). This could be viewed as an extension of the standard spin diffusion model^27^ which uses two separate electrochemical potentials for *U* and *D* states. Our model uses four: *U*+, *D*+, *U*−, and *D*−. Time reversal symmetry requires the number of transverse modes to be the same for *U*+ and *D*− states (*M*) and for *U*− and *D*+ states (*N*), as shown in Fig. 1(b). In principle, the ferromagnetic contact could break the time reversal symmetry of the underlying TI layer, which we have not considered in this paper. It is possible that future experiments with strongly coupled ferromagnetic contacts will require an extension of our model to include unequal number of modes for *U*+, *D*− and for *U*−, *D*+. However, some recent experiments^1^,^3^,^4^,^10^,^11^,^28^,^29^ show relative robustness of TI surface states in the presence of ferromagnetic contacts, where the effect of time reversal symmetry breaking appears minimal possibly because any modification of the TI band structure appears to occur around the Dirac point^28^,^29^.

## Parameters

Three parameters appear in our equivalent circuits, namely the channel polarization (*p*_0_), the ballistic conductance (*G*_*B*_) and the ordinary conductance (*G*) which are given by


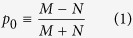



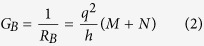



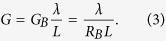


**Figure 1:**
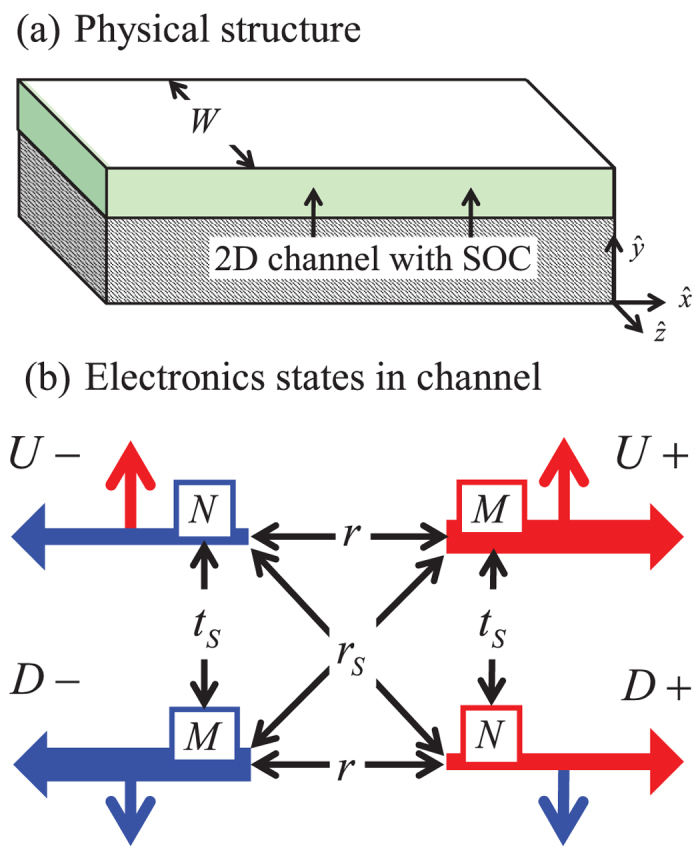
(**a**) Physical structure: A two dimensional (2D) channel with spin-orbit coupling (SOC). (**b**) Electronic states in the channel are classified into four groups depending on the sign of the *z*-component of the spin (up (*U*), down (*D*)) and the sign of the *x*-component of the velocity (+, −). Time reversal symmetry requires the number of transverse modes to be the same for *U*+ and *D*− states (*M*) and for *U*− and *D*+ states (*N*). Also indicated are three types of scattering rates per unit length, namely *r*, *r*_*s*_, and *t*_*s*_ corresponding to reflection without spin-flip, reflection with spin-flip and transmission with spin-flip respectively. For a more detailed discussion of *U*±, *D*± see Fig. 1 in the supplementary information.

We also define three scattering rates per unit length, namely *r*, *r*_*s*_, and *t*_*s*_ for three types of scattering processes, representing reflection without spin-flip, reflection with spin-flip and transmission with spin-flip respectively as indicated in Fig. 1(b). Correspondingly there are several distinct mean free paths e.g.


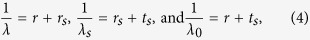


that appear in the full model, though only the first mean free path (*λ*) appears in the simple equivalent circuits that we present.

Although the ballistic conductance *G*_*B*_ appears as a parameter, our results are not limited to ballistic transport, and are valid in general from ballistic to diffusive regime. *G*_*B*_ simply represents a material parameter defined by Eq. (2). Supplementary information describes how the number of modes *M*, *N* are estimated for a given Hamiltonian. The concept of modes or channels plays a central role in mesoscopic physics and have a deeper significance beyond what the simple derivation might suggest^30^.

The key parameter here is the channel polarization *p*_0_ defined in terms of *M* and *N* (Eq. (1)): It provides a common link among diverse 2D channels with SOC. TI surface states represent a special case of this model with *N* = 0 thus providing the highest value of *p*_0_. In practice, however, parallel channels are usually present making the effective *N* greater than zero and lowering the effective *p*_0_. Note that *p*_0_ is well-defined not only for TI but also for more general cases like the Rashba Hamiltonian where it is energy-dependent and has direct physical interpretation and consequences^31^. In this paper we present an explicit relationship between the parameter *p*_0_ and Rashba/topological insulator channels, but similar expressions could be obtained for other mechanisms as well through an appropriate redefinition of the three parameters. Alternatively these parameters could be obtained directly from experiment without reference to any microscopic theory. The proposed circuit contains linear elements which with appropriate energy averaging can incorporate non-zero temperature related effects. However, at higher bias the circuit elements may need to be bias-dependent.

Our approach is based on a terminal description with the channel described by three physical parameters: *p*_0_, *G*_*B*_, and *G*. This terminal description is first obtained from heuristic arguments and then from a detailed semiclassical model based on the four electrochemical potentials mentioned earlier. There has been much discussion in the literature regarding subtle issues^32^ related to (a) the non-zero equilibrium spin currents and (b) the non-conservation of spin currents. Our model takes care of (a) by defining the spin current relative to that in the equilibrium state with a common electrochemical potential *μ*_*eq*_. This relative quantity allows us to extract circuit parameters needed to model non-equilibrium measurements. Regarding (b) our model includes it through scattering processes in Eq. (4) just as Valet-Fert equations included it through spin-flip processes. The model, however, misses any effect (e.g. spin precession) involving off-diagonal elements of the density matrix, which are presumed negligible due to phase breaking processes.

## Outline

**Figure 2:**
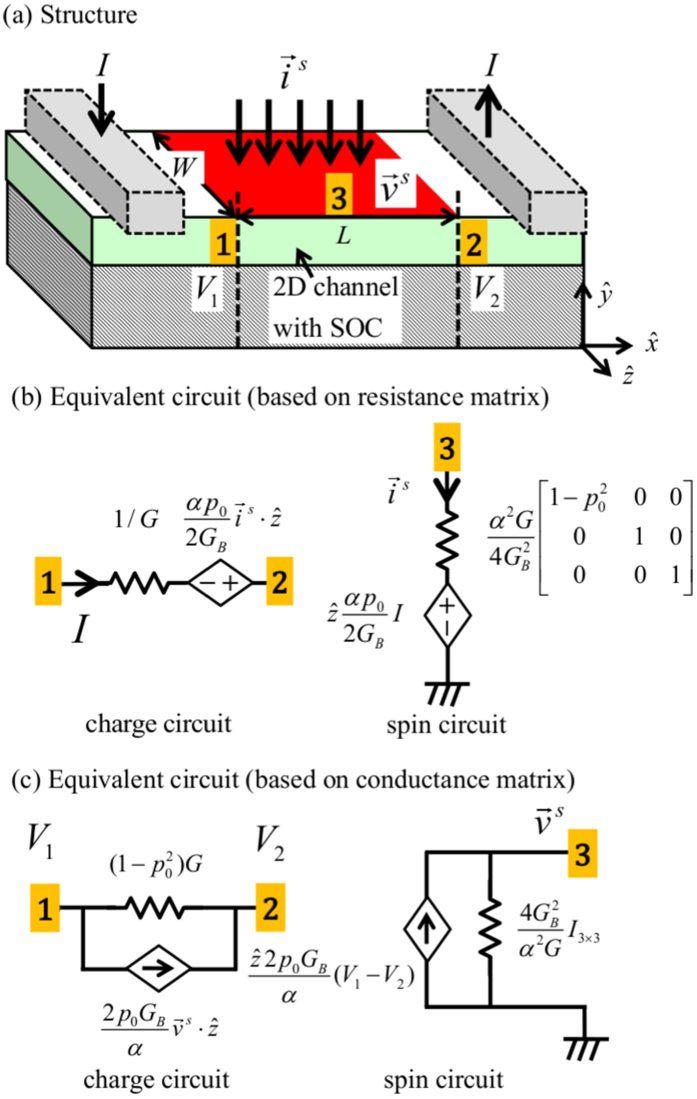
(**a**) Physical structure: A two dimensional (2D) channel with spin-orbit coupling (SOC) subject to a constant current *I* flowing along *x*-direction and a spin current 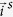 injected into the surface as shown. The overall system is treated as a three-terminal device with two charge terminals 1 and 2, and a spin terminal 3 with a three component spin voltage 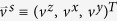 and current 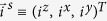 indicating the direction of the spin. (**b**) Equivalent circuit representation based on resistance matrix. (**c**) Equivalent circuit representation based on conductance matrix where *I*_3×3_ represents a 3 by 3 identity matrix. Parameters are defined in Eqs (1)–(3).

The outline of this paper is as follows. We first summarize the main results followed by an intuitive derivation in the heuristic derivation section. We then show that the predictions from the circuit model match quantitatively the results obtained from a full quantum transport model based on the non-equilibrium Green’s function (NEGF) formalism for a 1D channel including scattering processes. Then we present a semiclassical model that can be viewed as an extension of the usual spin diffusion equations to include four electrochemical potentials *U*+, *D*+, *U*−, and *D*− as described earlier. We use it to provide a formal justification of the equivalent circuits representing the structure in Fig. 2(a), but it should be noted that this approach can be used to treat more general contact structures beyond the one shown in Fig. 2(a). Indeed some readers may prefer to look at this formal derivation first, before looking at the more heuristic discussions in the earlier sections.

## Main Results

### [*R*] matrix

In an earlier paper^31^ it was shown that the flow of a current *I* along a channel with SOC leads to the generation of a surface spin voltage (Fig. 1(a)) given by


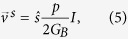


where 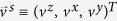 represents a three component spin voltage and 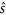 is a unit vector along the spin polarization direction.

A number of experimental observations^4^,^5^,^6^,^7^,^8^,^9^ have supported this result and one of the important objectives of this paper is to extend it to provide a description of processes that extract or inject a spin current 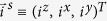 from or into the surface as shown in Fig. 2(a). Specifically we show that for a channel where reflection with spin flip is the dominant scattering process, the resistance matrix is given by


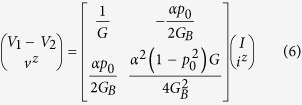


where *α* (0 ≤ *α* ≤ 1) is the angular averaging factor. This angular averaging factor comes from the fact that positive propagating states or modes have some angular variations depending on their eigenstates or detailed scattering processes. In the simplest approximation the angle *θ* between spin polarization and the *z*-axis varies from −*π*/2 to +*π*/2 as the angle of propagation (that is the *k*-vector) changes and so we need to average over *θ*, which will give *α* = 2/*π* with *p* = *αp*_0_. This resistance matrix can be translated into the equivalent circuit in Fig. 2(b). The conductance matrix is given by


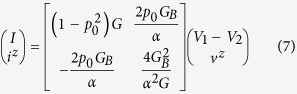


which can be translated into the equivalent circuit shown in Fig. 2(c) where other components of spin (*x*, *y*) are added with no coupling with charge (*p*_0_ = 0). Here 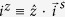 and 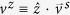.

Note that in Eq. (6) *R*(1, 2) = −*R*(2, 1) = *αp*_0_/2*G*_*B*_ as required by reciprocity^33^, the extra negative sign arising from the reversal of spin (but not charge) on reversing time. The *R*(2, 1) element in Eq. (6) represents our earlier result for the open circuit spin voltage corresponding to zero spin current 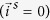. The *R*(1, 2) element represents an inverse effect similar to what has been described as the Rashba-Edelstein effect^18^,^34^, whereby an injected spin current causes a voltage to appear in the charge circuit.

The element *R*(1, 1) gives the ordinary resistance 1/*G* as we might expect. However, the element *R*(2, 2) is non-intuitive and extremely important since it determines the maximum spin current that can be extracted for a given charge current.

From Eqs (6) and (7) it is immediately clear that the measured conductance would change from *G* for a spin open circuit 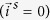 to 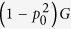 for a spin short circuit 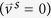. One way to go continuously from a spin open circuit to a spin short circuit is to use a magnetic insulator like YIG (yttrium iron garnet) and rotate its magnetization from the *z*-direction to the *x*-direction. SHE materials have been shown to exhibit the phenomenon of spin Hall magnetoresistance^15^,^35^. Our model suggests that a similar phenomenon should be observed for any 2D spin-orbit channel and the magnitude of the effect depends on the square of the channel polarization, *p*_0_. More general expressions of Eqs. (6) and (7) considering all scattering mechanisms are given in the semiclassical model section.

### Equivalent spin-Hall angle

Indeed as observed from the terminals, the effects described here for a 2D channel with SOC mimic those associated with the SHE which is commonly described in terms of a bulk spin Hall angle (*θ*_SHE_) for a sample of thickness *t*. For example, Eq. (6) suggests that with a very high spin conductive load 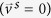 the ratio of the spin current to the charge current is given by


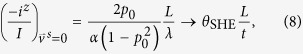


which can be equated to the standard expression for the SHE to obtain an effective spin Hall angle


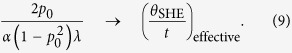


Not surprisingly, the effective spin Hall angle is related to the channel polarization *p*_0_, but a less intuitive prediction is that the backscattering length *λ* plays the role of the film thickness *t*: note that our 2D channel has no intrinsic thickness in the *y*-direction.

### Maximum spin current density

The previous result can also be used to obtain a simple estimate for the maximum spin current density that can be extracted from a 2D channel with SOC:


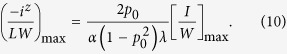


Assuming *p*_0_ ≈ 0.5 and *α* ~ 1 corresponding to 2D TI surface states with parallel channels, the maximum spin current density equals *I*/*Wλ*. The charge current per unit width is given by^36^


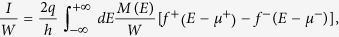


with *f*^+^(*E* − *μ*^+^) and *f*^−^(*E* − *μ*^−^) representing occupational factors for positive and negative propagating modes. Assuming (*μ*^+^ − *μ*^−^)_max_ ≈ *E*_*G*_ so that *f*^+^(*E* − *μ*^+^) ≈ 1 and *f*^−^(*E* − *μ*^−^) ≈ 0 over an energy range of *E*_*G*_ provides an estimation of maximum charge current per unit width at low temperature.


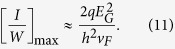


With a bandgap *E*_*G*_ ~ 0.5 eV and Fermi velocity *v*_*F*_ ~ 5 × 10^5^ ms^−1^, we have a maximum *I*/*W* ~ 10 mA/*μ*m, so that to obtain a spin current density of 10^6^ A/cm^2^ we need a mean free path less than 1 *μ*m.

A non-intuitive aspect of Eq. (9) is that one needs ***shorter*** mean free paths (*λ*) and hence ***higher resistivity*** in order to obtain a higher effective spin Hall angle and hence extract more spin current from a given structure. This seems similar to what is experimentally observed for materials with spin Hall effect: high resistivity phase of a given material shows larger spin Hall angles^13^,^14^.

## Heuristic Derivation

In this section we present an intuitive derivation of Eq. (6) which is represented by the circuit model shown in Fig. 2(b). The conductance matrix version in Eq. (7) and Fig. 2(c) then follows as a corollary.

The [*R*] matrix in Eq. (6) has four elements which appear as elements of the circuit in Fig. 2(b). *R*(1, 1) is just the ordinary resistance 1/*G*, with *G* given by Eq. (3). Below we will justify the elements *R*(2, 1) and *R*(2, 2). The remaining element *R*(1, 2) follows from *R*(2, 1) through reciprocity.

We first note that the charge current is given by the difference between those carried by the forward states (*U*+, *D*+) and the backward states (*U*−, *D*−)


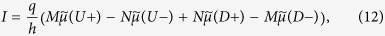


where electrochemical potentials 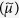 are defined relative to the equilibrium state (*μ*_*eq*_) i.e. 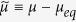 (see supplementary information for derivation).

The spin voltage is given by the difference between the weighted average of the up channels (*U*+ and *U*−) and that of the down channels (*D*+ and *D*−) (see supplementary information):


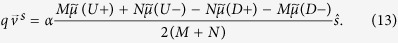


Here *α* is added to denote the angular averaging effect where only partial number of modes of *M* and *N* contribute for spin effectively.

**Figure 3:**
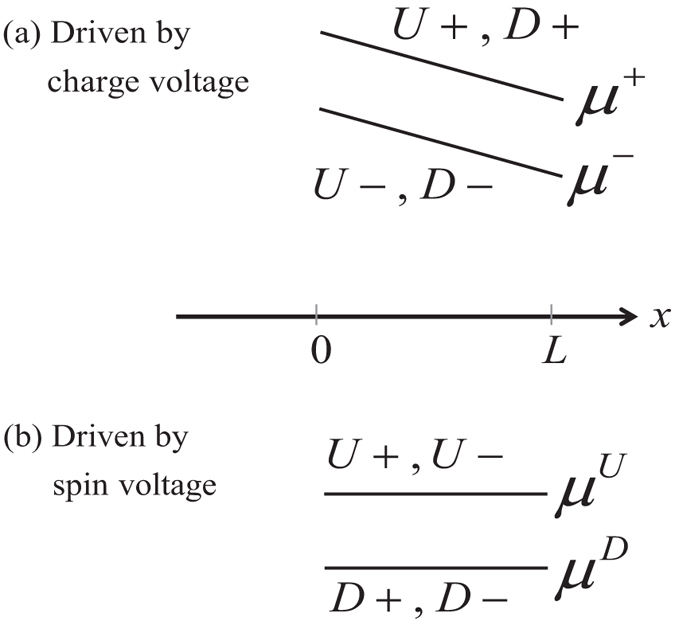
Two special cases considered in this Section to extract the coefficients of the *R*-matrix in Eq. (6). (**a**) Separate electrochemical potential profile for *U*+, *D*+ and *U*−, *D*− respectively in the channel driven by a charge voltage. (**b**) Separate electrochemical potential profile for *U*+, *U*− and *D*+, *D*− respectively in the channel driven by a spin voltage.

To obtain *R*(2, 1) we consider a special case where the channel is driven by a charge voltage along 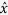 creating the potential profile shown in Fig. 3(a) with


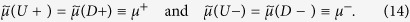


Using Eq. (14) in Eqs. (12) and (13) we have


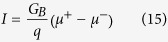



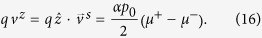


Combining Eqs (15) and (16) we have the result stated in the introduction *v*^*z*^ = *αp*_0_*I*/2*G*_*B*_ with *i*^*z*^ = 0 which leads to the stated value of *R*(2, 1) = *αp*_0_/2*G*_*B*_.

To obtain *R*(2, 2) we consider another special case where the channel is driven by a spin voltage creating the potential profile shown in Fig. 3(b) with


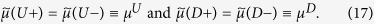


Using Eq. (17) in Eqs (12) and (13) we have


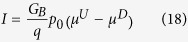



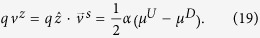


From Eq. (6) we have


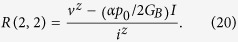


Since *V*_1_ − *V*_2_ = 0, we have *i*^*z*^ = (2*G*_*B*_/*αp*_0_*G*)*I* from Eq. (6) so that


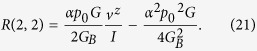


Using Eqs (18) and (19) we obtain the expression stated earlier, namely 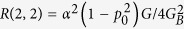.

## NEGF Benchmark

In this section we compare the predictions of the circuit model in Fig. 2 quantitatively with the results from a non-equilibrium Green’s function (NEGF) based model for a simple 1D TI surface states having *M* = 1, *N* = 0 so that *p*_0_ = 1. Note that in this case the factor *α* = 1 because angular averaging over transverse directions is absent. From the resistance matrix in Eq. (6) we can write


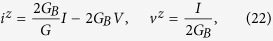


where *V* ≡ *V*_1_ − *V*_2_.

**Figure 4:**
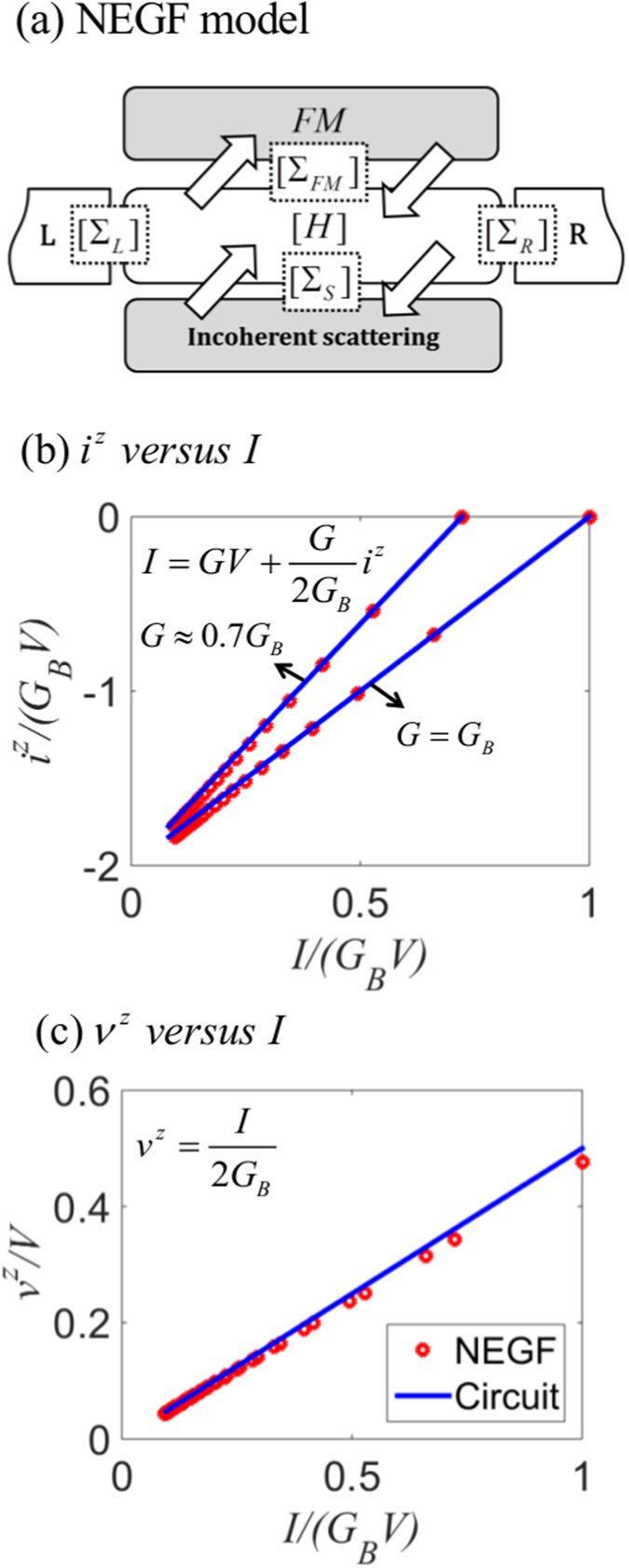
NEGF result compared with the proposed spin circuit for a 1D topological insulator (*P*_0_ = 1). (**a**) NEGF model: Hamiltonian (*H*) with four different self energies are shown. Σ_*L*_ and Σ_*R*_ are used for left and right contacts. Σ_*S*_ represents the incoherent scattering in the intrinsic 2D channel. Σ_*FM*_ represents the effect of a ferromagnet (FM) which is modeled as a spin mixing conductance with isotropic momentum relaxation scattering in real space. (**b**,**c**) Comparison of results from Eq. (6) (solid lines) and from NEGF (circles).

To test this prediction we use the NEGF model summarized in Fig. 4(a) with a Hamiltonian *H* and four different self energies: Σ_*L*_, Σ_*R*_ representing the left and right contacts, Σ_*S*_ representing the scattering processes in the channel and Σ_*FM*_ representing an external load that extracts a spin current 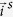 as shown in Fig. 2(a) (model details provided below).

As we vary the magnitude of Σ_*FM*_ with a fixed *V*, the current *I* changes along with the spin voltage 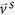 and the spin current 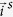. Figure 4(b,c) compares the variation of spin current and spin voltage against the charge current calculated from the NEGF model against the prediction of the circuit model (Eq. (22)), showing good agreement.

Below are the details of the model following the discussion and notation in ref. 31.

### Hamiltonian

The model Hamiltonian for topological insulator surface states (TISS) is given by


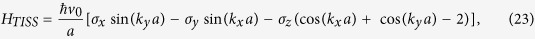


with *σ*_*x*_, *σ*_*y*_, *σ*_*z*_ the Pauli spin matrices and *a* the lattice spacing and *v*_0_ the Fermi velocity respectively.

### Self energy for contact

Two self energies Σ_*L*_ and Σ_*R*_ are used for left and right contacts representing semi-infinite contacts of extended channel.

### Self energy for incoherent scattering

The incoherent scattering in the channel is included by the self energy Σ_*S*_ with isotropic momentum and spin relaxation in the self-consistent Born approximation. The momentum randomizing scattering is described by^37^


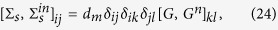


with *i*, *j*, *k*, and *l* representing indices in real space. The spin randomizing scattering is described by^37^


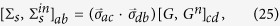


with *a*, *b*, *c*, and *d* representing indices in spin space.

### Self energy for FM

The self energy for FM ([Σ_*FM*_]) is modeled as an additional scattering process in the channel represented by isotropic momentum and spin relaxations in the self-consistent Born approximation.

### Currents and Voltages

The current operator at terminal “*i*” is defined as^36^


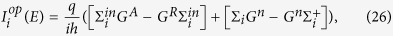


for a given energy. The charge and spin currents are calculated from


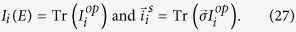


***Charge and spin occupation factors*** are calculated from


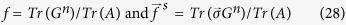


and to compare with the proposed circuit model the following identifications are made, which can be justified within a linear response regime,


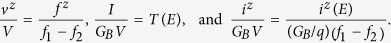


## Semiclassical Model

Here we provide a formal justification of the proposed circuit based on a semiclassical model. Starting from the steady-state Boltzmann equation


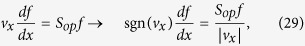


with *S*_*op*_ denoting a scattering operator we obtain after applying the relaxation time approximation


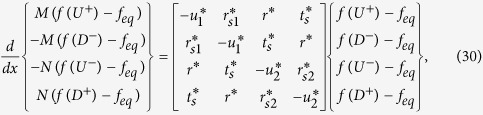


where u^*^_1_ = r^*^_s1_ + r^*^ + t^*^_s_ , u^*^_2_ = r^*^_s2_ + r^*^ + t^*^_s_ and *f*_*eq*_ represents the equilibrium Fermi function. We have chosen a set of parameters consistent with reciprocity, charge conservation, and the requirement of zero current for equal potentials. Using the linear response approximation for the Fermi function:


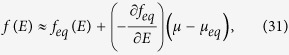


we obtain the following equation describing the spatial evolution of the electrochemical potentials for the four groups of states *U*+, *D*+, *U*−, and *D*−,


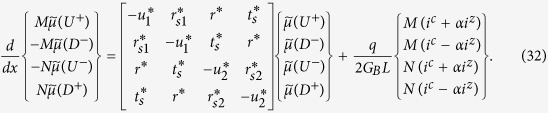


where 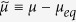 are the electrochemical potentials defined relative to the equilibrium state. Here we have added the last term to account for charge (*i^c^*) and spin (*i^z^*) currents injected from external sources and the factor a is the same as in Eq. (13) arising from the angular averaging of the spin direction associated with different modes.

We now transform Eq. (32) in terms of charge and spin voltages and currents in the channel defined as


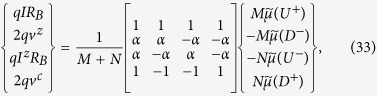


where the first two equations were introduced earlier (Eqs. (12), (13)), the last two follow similarly (see supplementary information for derivation). From Eqs. (32) and (33) we get using straightforward algebra (details in supplementary information)


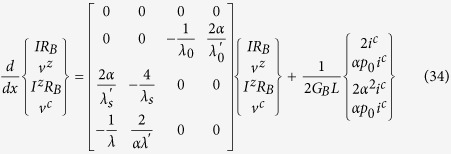


where λ, λ_*s*_, and λ_0_ are given by Eq. (4). λ′, λ′_*s*_ , and λ′_0_ are given by.


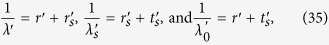


with


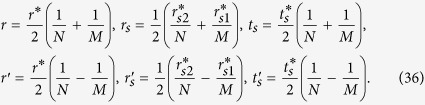


We will now specialize to problems for which there are no external charge current, *i^c^* =0 and we can assume that *dv^z^*/*dx* = 0: we then have from the second line of Eq. (34)


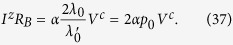


which when used with the last two lines of Eq.(34) gives


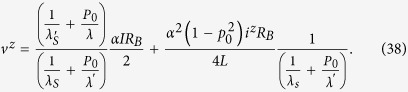


Combining the last equation of Eq. (34) with *dV^c^*/*dx* = -(*V*_1_ - *V*_2_)/*L* and Eq. (38), we have


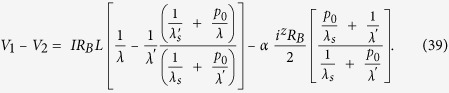


Eqs (38) and (39) can be rewritten in the following form


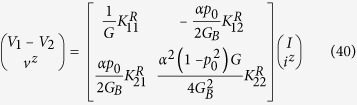


which represents the generalized version of Eq. (6) stated earlier, where *G* is defined inEq. (3) and *K^R^* denote the correction factors:


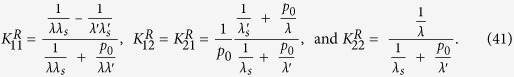


The corrections factors *K^R^* are ~1 if *r_s_* ≫ *r*, *t_s_*, *r_s_′* that is if reflection with spin fip is the dominant scattering process in the channel. Inverting Eq. (40) we obtain the generalized version of the conductance matrix in Eq. (7)


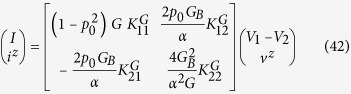


with correction factors *K*G** given by


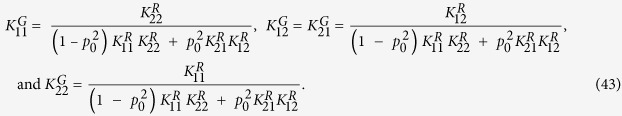


## Summary

A spin circuit model for 2D channels with spin-orbit coupling is proposed that can be used to interpret experimental results and estimate important quantities like the effective spin Hall angle, maximum spin current density and magnetoresistance. Some experimental support is already available^4^,^5^,^6^,^7^ and we hope more will be forthcoming. A heuristic justification as well as a semiclassical derivation is provided for the proposed circuit with an emphasis on the concept of propagating modes in the channel. Specifically, four types of modes depending on their spin (up and down) and propagating directions (positive and negative) are introduced together with chemical potentials for each of them. We also show with a simple 1D example that results from the circuit model agree well with those obtained from a quantum transport simulation based on nonequilibrium Green’s function (NEGF) model. We believe that the proposed spin circuit can be used to model simple structures (Fig. 2(a)) while the underlying semiclassical model can be used for more general contact structures^38^,^39^.

## Additional Information

**How to cite this article**: Hong, S. *et al.* Spin Circuit Model for 2D Channels with Spin-Orbit Coupling. *Sci. Rep.* **6**, 20325; doi: 10.1038/srep20325 (2016).

## Supplementary Material

Supplementary Information
