# Supplementary material for: Spin Circuit Model for 2D Channels with Spin-Orbit Coupling
Source: Sci Rep. 2016 Mar 2;6:20325. doi: 10.1038/srep20325 (PMC4773926; doi:10.1038/srep20325)
Supplement: Supplementary Information [file srep20325-s1.pdf]

# Spin Circuit Model for 2D Channels with Spin-Orbit Coupling: Supplementary Information

Seokmin Hong, Shehrin Sayed, and Supriyo Datta
*School of Electrical and Computer Engineering, Purdue University, IN, 47907*

## I. TRANSVERSE MODES

*This section outlines how the number of modes M, N in Fig. 1(b) of the main manuscript can be obtained for a given Hamiltonian.*

For a given Hamiltonian one can estimate $p_0$ based on the picture in Fig. 1(b). For example, in the case of TISS ($N = 0$) it is given by

$$p_0 = 1, \tag{I.1}$$

based on a following type of Hamiltonian

$$H = \hbar v_0 \left(\vec{\sigma} \times \vec{k}\right) \cdot \hat{n}. \tag{I.2}$$

Here $\hat{n}$ is an outward normal vector from the surface, $\vec{\sigma}$ is a vector of the Pauli spin matrices, $v_0$ is the Fermi velocity. For the channel with Rashba SOC ($E \geq 0$), it is given by

$$p_0(E) = \frac{k_2 - k_1}{k_2 + k_1} = \frac{1}{\sqrt{1 + 2E/(mv_0^2)}}, \tag{I.3}$$

with the following Hamiltonian

$$H = \frac{\hbar^2 k^2}{2m} I_{2\times 2} - \hbar v_0 \left(\vec{\sigma} \times \vec{k}\right) \cdot \hat{n}. \tag{I.4}$$

The polarization direction of spin in TISS and Rashba is determined by $\hat{s} = \text{sign}(\hbar v_0)\, \hat{I} \times \hat{n}$ with $\hat{n}$ being an outward normal vector and $\hat{I}$ being a charge current direction so that we have $\pm\hat{z} = \pm\hat{x} \times \hat{y}$ polarized spin in the structure of Fig. S1.

## II. CURRENTS AND VOLTAGES

*This section outlines how Eq. (33) in the main manuscript was derived for the 2D channel with spin-orbit coupling (SOC).*

### A. Charge Current

The semiclassical expression for charge current in the channel is given by

$$I = \frac{q}{h} \int dE \left[ M(E) \left( f\left(E - \mu\left(U+\right)\right) - f\left(E - \mu\left(D-\right)\right)\right) - N(E) \left( f\left(E - \mu\left(U-\right)\right) - f\left(E - \mu\left(D+\right)\right)\right)\right], \tag{II.1}$$

where $f(E) = 1/\left(1 + e^{E/kT}\right)$ is the Fermi function. Under the linear response approximation (see Eq. (31) in the main manuscript) for the Fermi function:

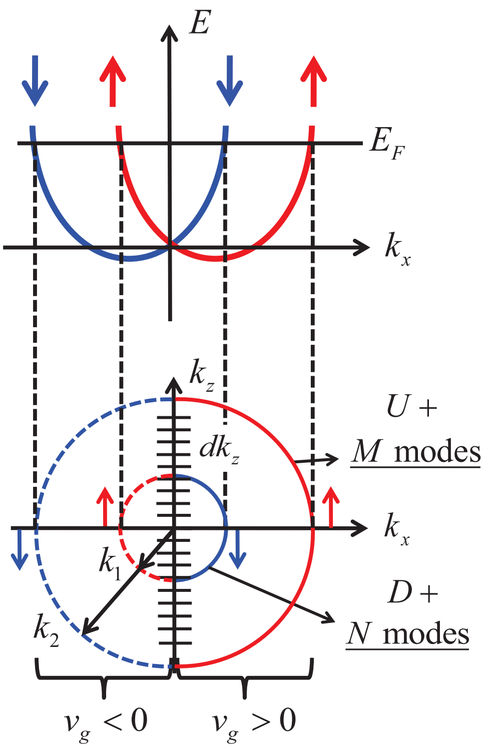


FIG. S1. Band diagram 2D channel with Rashba SOC is shown where two Fermi circles having opposite spin polarizations exist with radius $k_1$ and $k_2$ respectively at a given energy $E_F$. Here small arrows represent spin directions and right (left) half circles with solid (dotted) lines have positive (negative) group velocities, $v_g > 0$ ($v_g < 0$). The right and outer half circle corresponds to modes with positive group velocities and up spin ($U+$ with $M$ modes) and the right and inner half circle corresponds to modes with positive group velocities and down spin ($D+$ with $N$ modes).

$$f(E-\mu) \approx f_{eq}(E-\mu_{eq}) + \left(-\frac{\partial f_{eq}}{\partial E}\right)(\mu-\mu_{eq}) \tag{II.2}$$

where $\mu_{eq}$ is the equilibrium electrochemical potential. Under this approximation Eq. (II.1) becomes

$$I = \frac{q}{h}\left(M\tilde{\mu}\,(U+) - N\tilde{\mu}\,(U-) + N\tilde{\mu}\,(D+) - M\tilde{\mu}\,(D-)\right),$$

which gives Eq. (12) and first line of Eq. (33) in the main manuscript, where we defined

$$\tilde{\mu} = \mu - \mu_{eq}, \tag{II.3}$$

$$M = \int dE \left(-\frac{\partial f_{eq}}{\partial E}\right) M(E), \text{ and } N = \int dE \left(-\frac{\partial f_{eq}}{\partial E}\right) N(E). \tag{II.4}$$

### B. Spin Voltage

The spin voltage in the channel is given by

$$q\,\vec{v}^s = \alpha\frac{M\mu\,(U+) + N\mu\,(U-) - N\mu\,(D+) - M\mu\,(D-)}{2\,(M+N)}\hat{s}. \tag{II.5}$$

Subtracting each electrochemical potential by $\mu_{eq}$ according to Eq. (II.3) gives second line of Eq. (33) in the main manuscript, noting that $v^z \equiv \hat{z}\cdot\vec{v}^s$.

$$q\,\vec{v}^s = \alpha\frac{M\tilde{\mu}(U+) + N\tilde{\mu}(U-) - N\tilde{\mu}(D+) - M\tilde{\mu}(D-)}{2\,(M+N)}\hat{s}.$$

### C. Spin Current

The definition of spin current in the channel based on our four electrochemical potentials (see Fig. 1(b) in main manuscript) is straightforward and includes both equilibrium and non-equilibrium conditions.

$$\tilde{I}^s = \alpha\frac{q}{h}\int dE\,[M(E)\,(f\,(E-\mu\,(U+)) + f\,(E-\mu\,(D-))) - N(E)\,(f\,(E-\mu\,(U-)) + f\,(E-\mu\,(D+)))]\,\hat{s}, \tag{II.6}$$

Under the linear response approximation (Eq.(II.2)), we can write Eq.(II.6) as

$$\tilde{I}^s = \alpha\frac{2q}{h}\int dE\;[M(E)-N(E)]\;f_{eq}(E-\mu_{eq})\hat{s} \,+\, \alpha\frac{q}{h}\,(M\tilde{\mu}\,(U+) - N\tilde{\mu}\,(U-) - N\tilde{\mu}\,(D+) + M\tilde{\mu}\,(D-))\,\hat{s}, \tag{II.7}$$

where $M$ and $N$ are defined by Eq.(II.4). The first part of the Eq.(II.7) gives the equilibrium spin current in the channel and the second part gives the non-equilibrium spin current in the channel, $z$-component of which is the third line of Eq.(33) in the main manuscript. Note that at equilibrium condition i.e. $\mu(U+) = \mu(U-) = \mu(D+) = \mu(D-) = \mu_{eq}$, third line of Eq.(33) in the main manuscript gives zero spin current.

### D. Charge Voltage

The charge voltage in the channel is given by

$$q\,\tilde{V}^c = \frac{M\mu(U+) + N\mu(U-) + N\mu(D+) + M\mu(D-)}{2\,(M+N)}. \tag{II.8}$$

The fourth line of Eq.(33) in the main manuscript is given in terms of $\tilde{\mu}$ as

$$q\,\tilde{V}^c = \frac{M\tilde{\mu}(U+) + N\tilde{\mu}(U-) + N\tilde{\mu}(D+) + M\tilde{\mu}(D-)}{2\,(M+N)} + \mu_{eq} = qV^c + \mu_{eq}.$$

Note that the equilibrium part in charge voltage gives a constant shift which cancels out in our model as we take the difference of charge voltages across the channel.

### E. Derivation of Eq. (34)

Differentiating Eq. (33) in the main manuscript with respect to $x$ and combining it with Eq. (32) in the main manuscript gives

$$\frac{d}{dx}\left\{\begin{array}{l} qIR_B \\ 2qV_s \\ qI_sR_B \\ 2qV_c \end{array}\right\} = \frac{2}{M+N}\left[\begin{array}{cccc} 0 & 0 & 0 & 0 \\ -\alpha\left(r^*+t_s^*\right) & -\alpha\left(r^*+t_s^*\right) & \alpha\left(r^*+t_s^*\right) & \alpha\left(r^*+t_s^*\right) \\ -\alpha\left(r_{s1}^*+t_s^*\right) & \alpha\left(r_{s1}^*+t_s^*\right) & -\alpha\left(r_{s2}^*+t_s^*\right) & \alpha\left(r_{s2}^*+t_s^*\right) \\ -\left(r_{s1}^*+r^*\right) & \left(r_{s1}^*+r^*\right) & \left(r_{s2}^*+r^*\right) & -\left(r_{s2}^*+r^*\right) \end{array}\right]\left\{\begin{array}{l} \tilde{\mu}(U^+) \\ \tilde{\mu}(D^-) \\ \tilde{\mu}(U^-) \\ \tilde{\mu}(D^+) \end{array}\right\} + \frac{q}{G_BL}\left\{\begin{array}{l} i_c \\ \alpha p_0 i_c \\ \alpha^2 i_s \\ \alpha p_0 i_s \end{array}\right\}. \tag{II.9}$$

Inverting Eq. (33) in the main manuscript gives

$$\left\{\begin{array}{l} \tilde{\mu}(U^+) \\ \tilde{\mu}(D^-) \\ \tilde{\mu}(U^-) \\ \tilde{\mu}(D^+) \end{array}\right\} = \frac{M+N}{4}\left[\begin{array}{cccc} \frac{1}{M} & \frac{1}{\alpha M} & \frac{1}{\alpha M} & \frac{1}{M} \\ -\frac{1}{M} & -\frac{1}{\alpha M} & \frac{1}{\alpha M} & \frac{1}{M} \\ -\frac{1}{N} & \frac{1}{\alpha N} & -\frac{1}{\alpha N} & \frac{1}{N} \\ \frac{1}{N} & -\frac{1}{\alpha N} & -\frac{1}{\alpha N} & \frac{1}{N} \end{array}\right]\left\{\begin{array}{l} qIR_B \\ 2qV_s \\ qI_sR_B \\ 2qV_c \end{array}\right\}. \tag{II.10}$$

Combining Eqs. (II.9) and (II.10) yields Eq. (34) in the manuscript with $(\lambda, \lambda')$ , $(\lambda_s, \lambda'_s)$, and $(\lambda_0, \lambda'_0)$ given by Eqs. (4), (35), and (36) in the main manuscript.
